# Immune checkpoint inhibitor-induced aplastic anaemia: Case series and large-scale pharmacovigilance analysis

**DOI:** 10.3389/fphar.2023.1057134

**Published:** 2023-01-26

**Authors:** Qian Guo, Jin Ning Zhao, Ting Liu, Jian Gao, Hui Guo, Jing Min Cheng

**Affiliations:** ^1^ School of Public Health, Shanxi Medical University, Taiyuan, Shanxi, China; ^2^ Department of Pharmacy, Second Hospital of Shanxi Medical University, Taiyuan, Shanxi, China; ^3^ School of Management, Shanxi Medical University, Taiyuan, Shanxi, China; ^4^ Department of Pharmacy, Shanxi Cardiovascular Disease Hospital, Taiyuan, Shanxi, China

**Keywords:** immune checkpoint inhibitors, aplastic anaemia, immune-related adverse events, food and drug administration’s adverse event reporting system, data mining

## Abstract

**Introduction:** Impressive advances in immunotherapy especially immune checkpoint inhibitors have made great progress in treating multiple cancers but can also cause serious even incurable immune-related adverse events, mostly found in colitis, dermatitis, hepatitis, and thyroiditis patients. Rare autoimmune hematologic toxicities have been reported in the literature, but are poorly described. Aplastic anaemia induced by immune checkpoint inhibitors is a life-threatening autoimmune disease; however, only a few cases have been reported in the literature.

**Objective:** To characterize and evaluate Aplastic anaemia associated with different ICI regimens in public database and review the literature.

**Methods:** We described a case series of patients experiencing Aplastic anaemia while on immune checkpoint inhibitors. We also mined the Food and Drug Administration’s Adverse Event Reporting System and used reporting odds ratio, the proportional reporting ratio, the Bayesian confidence propagation neural network and the multi-item gamma Poisson shrinker algorithms to achieve the data of the suspected adverse events of Aplastic anaemia-induced by immune checkpoint inhibitors between January 2011 and June 2022.

**Results:** Thirteen patients with Aplastic anaemia events while on immune checkpoint inhibitors were included in our case series, and seven of them had a fatal outcome. In FAERS, a total of 38 individual case safety reports (immune checkpoint inhibitors) with different ICI regimens were retrieved, of which 25 (65.79%) were reported as monotherapy and 13 (34.2%) had a fatal outcome. The reporting odds ratio was significant for nivolumab (reporting odds ratio 3.05, 95%CI 1.73–5.38), pembrolizumab (reporting odds ratio 2.33, 95%CI 1.16–4.67), avelumab (reporting odds ratio 12.63, 95%CI 3.15–50.62) and ipilimumab/nivolumab (ROR 2.57, 95%CI 1.15–5.72).

**Conclusion:** There is a significant reporting signal of Aplastic anaemia with several ICI agents. Clinicians should raise awareness and monitor this potentially fatal adverse event.

## 1 Introduction

Since the first immune-checkpoint inhibitor (ICI) ipilimumab has been approved in the United States in 2011, cancer treatment has changed dramatically. The clinical outcomes of various types of cancer have been gradually improving and ICIs become a new pillar of precision cancer medicine (Nakajima and Nakatsura, 2021). ICIs, including programmed cell death protein-1 (PD-1) inhibitors, programmed death-ligand 1 (PD-L1) inhibitors and cytotoxic T-cell-associated protein-4 (CTLA-4) inhibitors, have exhibited promising antitumor efficacy in patients with the late malignant tumor for the past decade (Naimi et al., 2022). However, the widespread application of ICIs is inevitably accompanied by immune-related adverse events (irAEs), impairing several organ systems, especially the gastrointestinal tract, endocrine, skin, liver, lungs, and joints (Liu et al., 2021). Overall, the reported incidence of serious irAEs is approximately 13% with ICI monotherapy (Brahmer et al., 2018), and fatal irAEs have been reported in up to 0.3%–1.3% of patients receiving these agents (Wang et al., 2018).

Although rare, ICI therapy can lead to autoimmune hematologic irAEs (Haem-irAEs), including autoimmune hemolytic anemia, pure red cell aplasia, hemophagocytic lymphohistiocytosis, immune thrombocytopenia, and aplastic anemia (Davis et al., 2019). Hematological irAEs (Haem-irAEs) especially aplastic anemia (AA) related to immunotherapy have not been extensively characterized. Case reports of AA-induced by ICIs have been reported sporadically (Helgadottir et al., 2017; Michot et al., 2017; Meyers et al., 2018; Cheng and Jackson, 2019; Comito et al., 2019; Filetti et al., 2019; Ni et al., 2019; Rouvinov et al., 2019; Goda et al., 2021; Younan et al., 2021; Ghanem et al., 2022). However, none of the case series has evaluated more than 3 cases; thus, little is known about the timing, spectrum, and clinical manifestations of AA events induced by ICI. In this study, we review the published literature and queried the United States Food and Drug Administration Adverse Event Reporting System (FAERS) about the occurrence of AA after the administration of ICIs to provide a comprehensive clinical description of the AA-induced by ICI to date and to learn whether there is a safety signal between AA and ICI therapy.

## 2 Methods

### 2.1 Case series

We performed a literature search by Google Scholar and PubMed focusing on publications in English before 20 September 2022. The following search terms were used: (immune checkpoint inhibitors OR checkpoint inhibitor therapy OR checkpoint inhibitor treatment OR ipilimumab OR pembrolizumab OR atezolizumab OR nivolumab OR durvalumab OR avelumab OR cemiplimab) AND (aplastic anaemia) AND (case report OR case series). Eligibility criteria included any case report or case series describing aplastic anaemia irAEs developing during and likely related to ICI therapy. Patient information including age, gender, cycles of ICI regimen, aplastic anaemia treatment and outcome were collected from the review of medical records.

### 2.2 Phamacovigilance analysis

This real-world, retrospective, pharmacovigilance study is based on individual case safety reports (ICSRs) reported in the FAERS database. FAERS contains information on adverse events, medication error and product quality complaints resulting in adverse events submitted to the FDA. The FAERS database is designed to support the FDA’s post-marketing safety surveillance program for drug and therapeutic biologic products. And it is a classic spontaneous reporting system, which collects data including demographics, drugs, indications, outcomes, reactions, sources, and therapies. Data on ICSRs with the ICIs as the suspected drugs were collected from the FAERS database between January 2011 and June 2022. Adverse events in the FAERS database are coded using the preferred terms (PTs) in the Medical Dictionary for Regulatory Activities (MedDRA). Preferred Terms “aplastic anaemia” and “autoimmune aplastic anaemia” were used to identify aplastic anaemia cases related to ICIs. Adverse events were further reviewed to remove potential duplicates (ie, records that overlapped at least three in four key fields: event date, age, sex, and reporter’s country).

### 2.3 Statistical analysis

The clinical characteristics of age, sex, primary source, outcomes, reported year, source region, and indication were provided separately for each ICI regimen. According to the disproportionality analysis and Bayesian analysis, the reporting odds ratio (ROR), the proportional reporting ratio (PRR), the Bayesian confidence propagation neural network (BCPNN), and the multi-item gamma Poisson shrinker (MGPS) algorithms were applied to detect an association between different ICI regimens and AA events. The equations and criteria for the above four algorithms (Chen et al., 2020) are shown in Table 1. If one of the four algorithms met the criteria, a positive signal of AA was generated. When using the full database as a comparator, the above four parameters were calculated, however, only ROR was calculated for comparing different drug regimen subgroups (Salem et al., 2018). We used the Chi-square test for comparisons of categorical variables (fatal cases VS. non-fatal cases). The normally distributed and not normally distributed continuous variables were analyzed using the *t*-test and non-parametric tests, respectively. The statistical significance was determined at *p* < 0.05 with 95% confidence intervals. The analyses were conducted using the SPSS 23.0 statistical software.

**TABLE 1:** Summary of major algorithms used for signal detection.

Algorithms	Equation	Criteria
ROR	ROR=ad/bc	95%CI > 1, N ≥ 2
	95%CI=elnROR±1.961/a+1/b+1/c+1/d^0.5	
PRR	$PRR=a\left(c+d\right)/c/\left(a+b\right)$	PRR≥2, χ^2^ ≥ 4, N ≥ 3
	χ2=ad−bc^2a+b+c+d/a+bc+da+cb+d	
BCPNN	$IC=\log_{\;2}^{\;a\left(a+b+c+d\right)\left(a+c\right)\left(a+b\right)}$	IC025 > 0
	IC025=elnIC−1.961/a+1/b+1/c+1/d^0.5	
MGPS	$EBGM=a\left(a+b+c+d\right)/\left(a+c\right)/\left(a+b\right)$	EBGM05 > 2, N > 0
	EBGM05=elnEBGM−1.641/a+1/b+1/c+1/d^0.5	

Abbreviations: a: the number of reports with suspect adverse drug event (ADE) of the suspect drug; b: the number of reports with all other ADEs, of the suspect drug; c: the number of reports with the suspect ADE, of all other drugs; d: the number of reports with all other ADEs, of all other drugs; ROR: reporting odds ratio; CI: confidence interval; N: the number of co-occurrences; PRR: proportional reporting ratio; χ^2^: chi-squared; BCPNN: bayesian confidence propagation neural network; IC: information component; IC025: the lower limit of the 95% two-sided CI, of the IC; MGPS: multi-item gamma Poisson shrinker; EBGM: empirical Bayesian geometric mean; EBGM05: the lower 95% one-sided CI, of EBGM.

## 3 Results

### 3.1 Case series

Thirteen patients developed new aplastic anaemia with ICI monotherapy or combination therapy (Table 2). The median age at the time of development of the AA was 67 years (interquartile range (IQR) 45–78 years), and 7 (53.8%) were females. PD-1 inhibitors (nivolumab or pembrolizumab) were implicated in 10 (76.9%) patients and PD-1/CTLA4 combination therapy (nivolumab and ipilimumab) in 3 (23.1%) patients. The most common malignancies were NSCLC (n = 6, 46.1%) and metastatic melanoma (n = 4, 30.7%). The median time to onset was 4 ICI treatment cycles (IQR 1 treatment cycle to 20 months). Seven patients (53.8%) of the AA were detected on regular laboratory tests. Eleven of thirteen patients were treated with steroids, from which 7 patients did not respond to steroids and then died, while four responded well to steroids and three got a full recovery. Two patients had steroids and immunosuppressive therapy and still died from AA. Of all patients, nine patients had a fatal outcome (69.2%).

**TABLE 2:** Summary of case reports of immune checkpoint inhibitor-induced aplastic anaemia reported in the literature.

Reference	Age	Sex	Malignancy	ICI	Cycles of ICI	Treatment	Outcome
1 (Michot et al., 2017)	73	F	NSCLC	Nivolumab	12	IGIV, RBC and platelets transfusions, antibiotics	No response to IGIV; death at 1 month from febrile neutropenia
2 (Michot et al., 2017)	70	M	NSCLC	Nivolumab	10	Prednisone 1 mg/kg, norethandrolone, GCSF, RBC and platelets transfusions	Partial and transient response to Steroids; persistent pancytopenia still ongoing at 4 months
3 (Michot et al., 2017)	78	M	NSCLC	Nivolumab	1	Prednisolone 1 mg/kg, IGIV, GCSF, RBC, antibiotics and platelets transfusions	No response to steroids and IGIV; death at 3 months from the acute coronary syndrome
4 (Filetti et al., 2019)	63	F	NSCLC	Nivolumab	2	Dexamethasone 12 mg/daily	No response to steroids; death at 2 weeks from complications of pneumonia
5 (Rouvinov et al., 2019)	74	F	Metastatic melanoma	Nivolumab	4	Prednisone 1.5 mg/kg, eltrombopag 50–100 mg/day, blood and platelet transfusions	No response to steroids; death from aplastic anemia
6 (Cheng and Jackson, 2019)	61	M	AML	Nivolumab	11	Methylprednisolone 2 mg/kg/day	Good response to steroids; complete remission was achieved
7 (Comito et al., 2019)	57	F	Glioblastoma multiforme	Nivolumab	2	RBC and platelet transfusions, G-CSF, eltrombopag 50–100 mg	Death at 73 days after the second dose of nivolumab from aplastic anemia
8 (Ghanem et al., 2022)	72	F	NSCLC	Nivolumab	20 months	prednisone 1 mg/kg, cyclosporine 5 mg/kg bid, pegflgrastim 6 mg, eltrombopag 50 mg	No response to steroids and immunosuppressive therapy; death from shock
9 (Ni et al., 2019)	67	M	Metastatic melanoma	Pembrolizumab	8	IGIV, prednisone 1 mg/kg/day	Good response to steroids; recovered 2 months later
10 (Goda et al., 2021)	77	F	NSCLC	Pembrolizumab	1	Platelet transfusion, prednisolone 40 mg, TPO-ras and eltrombopag, G-CSF, danazol 300 mg	No response to steroids; good response to danazol; death at 3 months from cancer
11 (Helgadottir et al., 2017)	48	F	Metastatic melanoma	Ipilimumab/nivolumab	5	Prednisone 1 mg/kg, G-CSF, tranexamic acid, platelet transfusions	No response to steroids; death on day 11 of hospitalization from intracerebral hemorrhage
12 (Meyers et al., 2018)	51	M	Metastatic melanoma	Ipilimumab/nivolumab	2	Methylprednisone 70 mg/Day, RBC transfusion	Good response to steroids; complete remission was achieved
13 (Younan et al., 2021)	45	M	Metastatic renal cell carcinoma	Ipilimumab/nivolumab	2	Corticosteroid 1–2 mg/kg/day, IGIV, G-CSF, horse ATG 20 mg/kg, eltrombopag 150 mg tablet/daily and cyclosporine 6 mg/kg/day	No response to steroids and immunosuppressive therapy; death at 2 weeks from aplastic anemia

Abbreviations: NSCLC, non-small cell lung cancer; IVIG, intravenous immunoglobulin; RBC, red blood cells; G-CSF, granulocyte-colony stimulating factor; AML, acute myeloid leukemia; TPO, thrombopoietin receptor agonists; ATG, anti-thymocyte globulin.

### 3.2 Descriptive analysis from FAERS

Overall, 38 reports related to AA induced by ICIs were archived in the FAERS database from January 2011 and June 2022, of which 25 (65.8%) reported one ICI as a suspected drug. Explicitly, 12 (31.6%) ICSRs were attached to nivolumab as well as ipilimumab/nivolumab, 8 (21.1%) to pembrolizumab, 2 (5.3%) to atezolizumab and avelumab, 1 (2.6%) to durvalumab and atezolizumab/nivolumab. ICSRs of other ICI regimens were not discovered. The demographic and clinical characteristics of all ICSRs are listed in Table 3. The median age of patients of all ICSRs was 68 years (IQR 26–83, n = 29), and the median age of ICIs other than atezolizumab was similar. The majority of all ICSRs were female patients (57.9%) and all cases were classified as serious. Most of the cases (86.8%) were submitted by healthcare professionals and were from North America (44.7%) and Europe (31.6%). Other outcomes (36.8%) were the most prevalent outcome, followed by death (34.2%), hospitalization (21.1%) and life-threatening (7.9%). Lung cancer (31.6%) is the most common indication of these ICSRs, then skin cancer (26.3%). The reported year was basically distributed evenly between 2016–2022 except for 2018. These AA events occurred early after ICI initiation, with a median time to onset of 34 days (IQR 10–510, *n* = 11) for all ICSRs, and most ICSRs (72.7%) were collected within the first 2 months. Furthermore, no demographic differences existed between fatal and non-fatal cases except clinical outcomes (Supplementary Table S1).

**TABLE 3:** Clinical characteristics of patients with immune checkpoint inhibitor-induced aplastic anaemia collected from the FAERS database (January 2011 to June 2022).

Variables	Nivolumab n = 12	Pembrolizumab n = 8	Durvalumab n = 1	Atezolizumab n = 2	Avelumab n = 2	Ipilimumab + nivolumab n = 12	Atezolizumab + nivolumab n = 1	Total n = 38
Age median (range)	60 (26–75) n = 7	74 (58–81) n = 8	-	34 (34–34) n = 1	68.5 (68–69) n = 2	67 (48–83) n = 10	70 (70–70) n = 1	68 (26–83) n = 29
Sex
Male	2 (16.7)	2 (25.0)	-	1 (50.0)	1 (50.0)	3 (25.0)	1 (100.0)	10 (26.3)
Female	7 (58.3)	6 (75.0)	-	1 (50.0)	1 (50.0)	7 (58.3)	-	22 (57.9)
Not reported	3 (25.0)	-	1 (100.0)	-	-	2 (16.7)	-	6 (15.8)
Primary source								
Healthcare professional	11 (91.7)	6 (75.0)	1 (100.0)	2 (100.0)	2	11 (91.7)	-	33 (86.8)
Consumer	1 (8.3)	2 (25.0)	-	-	-	1 (8.3)	1 (100.0)	5 (13.2)
Outcomes								
Hospitalization	1 (8.3)	3 (37.5)	-	-	-	4 (33.3)	-	8 (21.1)
Life-threatening	1 (8.3)	1 (12.5)	-	-	-	1 (8.3)	-	3 (7.9)
Death	5 (41.7)	2 (25.0)	-	1 (50.0)	-	5 (41.7)	-	13 (34.2)
Other	5 (41.7)	2 (25.0)	1 (100.0)	1 (50.0)	2 (100.0)	2 (16.7)	1 (100.0)	14 (36.8)
Reported year								
2016	2 (16.7)	-	-	-	-	1 (8.3)	-	3 (7.9)
2017	1 (8.3)	-	-	1 (50.0)	-	-	-	2 (5.3)
2018	5 (41.7)	1 (12.5)	-	-	-	6 (50.0)	-	12 (31.6)
2019	2 (16.7)	1 (12.5)	-	-	-	2 (16.7)	1 (100.0)	6 (15.8)
2020	-	2 (25.0)	-	1 (50.0)	1 (50.0)	1 (8.3)	-	5 (13.2)
2021	2 (16.7)	2 (25.0)	1 (100.0)	-	1 (50.0)	-	-	6 (15.8)
2022	-	2 (25.0)	-	-	-	2 (16.7)	-	4 (10.5)
Source region								
North America	7 (58.3)	3 (37.5)	-	1 (50.0)	-	6 (50.0)	-	17 (44.7)
Europe	4 (33.3)	2 (25.0)	-	1 (50.0)	2 (100.0)	2 (16.7)	1 (100.0)	12 (31.6)
Asia	1 (8.3)	3 (37.5)	1 (100.0)	-	-	4 (33.3)	-	9 (23.7)
Indication								
Central nervous system tumor	3 (25.0)	-	-	-	-	-	-	3 (7.9)
Gastrointestinal cancer	1 (8.3)	1 (12.5)	-	-	-	-	-	2 (5.3)
Tumors of the female reproductive organs	-	-	-	1 (50.0)	-	-	-	1 (2.6)
Hematological cancer and lymphoma	1 (8.3)	1 (12.5)	-	-	-	-	-	2 (5.3)
Skin cancer	3 (25.0)	-	-	-	1 (50.0)	6 (50.0)	-	10 (26.3)
Lung cancer	1 (8.3)	5 (62.5)	1 (100.0)	-	-	5 (41.7)	-	12 (31.6)
Tumors of the urinary system	-	1 (12.5)	-	-	-	1 (8.3)	-	2 (5.3)
Unknown or missing	3 (25.0)	-	-	1 (50.0)	1 (50.0)	-	1 (100.0)	6 (15.8)
Time to onset, days	36 (36–36) n = 1	31.5 (10–510) n = 4	101 (101–101) n = 1	41 (41–41) n = 1	408 (408–408) n = 1	12 (11–27) n = 3	-	34 (10–510) n = 11

### 3.3 Signal values associated with different immunotherapy regimens

We detected signals of AA effects for all 5 ICI monotherapies (nivolumab, pembrolizumab, durvalumab, atezolizumab and avelumab) and 2 ICI combination therapies (ipilimumab/nivolumab and atezolizumab/nivolumab) using the criteria for the four algorithms and listed the results in Table 4. Among all ICI therapies, only the combination regimen of ipilimumab and nivolumab met all four criteria. The ROR was significant for nivolumab (ROR 3.05, 95%CI 1.73–5.38), pembrolizumab (ROR 2.33, 95%CI 1.16–4.67), avelumab (ROR 12.63, 95%CI 3.15–50.62). Among all ICI monotherapies, avelumab was particularly noteworthy for the relationship to AA events due to its highest ROR and EBGM. For the therapies of dual regimens, the combined ICIs of ipilimumab plus nivolumab appeared stronger associations with AA events than the single regimen of nivolumab, based on the higher ROR (ROR 2.57, 95%CI 1.15–5.72).

**TABLE 4:** Associations of different ICI regimens with aplastic anaemia.

ICI regimens	N	ROR (95% CI)	PRR (χ^2^)	IC (IC025)	EBGM (EBGM05)
Total	38	3.53 (2.56, 4.88)	3.53 (67.29)	54.78 (39.70)	3.47 (2.65)
Anti-PD-1	20	2.72 (1.75, 4.22)	2.72 (21.42)	53.29 (34.28)	2.69 (1.86)
Nivolumab	12	3.05 (1.73, 5.38)	3.05 (16.39)	51.65 (29.26)	3.03 (1.89)
Pembrolizumab	8	2.33 (1.16, 4.67)	2.33 (6.04)	50.86 (25.39)	2.32 (1.30)
Anti-PD-L1	5	2.67 (1.11, 6.42)	2.67 (5.19)	49.31 (20.49)	2.66 (1.28)
Durvalumab	1	2.87 (0.40, 20.37)	2.87 (1.21)	44.56 (6.27)	2.86 (0.56)
Atezolizumab	2	1.46 (0.37, 5.85)	1.46 (0.29)	47.53 (11.87)	1.46 (0.46)
Avelumab	2	12.63 (3.15, 50.62)	12.60 (21.34)	44.42 (11.08)	12.59 (3.94)
Ipilimumab + nivolumab	12	7.85 (4.45, 13.86)	7.84 (71.06)	50.29 (28.48)	7.79 (4.84)
Atezolizumab + nivolumab	1	101.13 (13.99, 731.29)	99.35 (97.31)	39.44 (5.45)	99.28 (18.97)
Ipilimumab/nivolumab vs nivolumab	2.57 (1.15, 5.72)				

Abbreviations: ICI: immune checkpoint inhibitor; N: the number of reports of ICI-associated aplastic anaemia; ROR: reporting odds ratio; CI: confidence interval; PRR: proportional reporting ratio; χ^2^: chi-squared; IC: information component; EBGM: empirical Bayes geometric mean.

The signal values which met criteria are marked in bold.

## 4 Discussion

To the best of our knowledge, this is the first analysis to utilize a pharmacovigilance method to explore the correlation between ICI treatment and increased risk of AA. The reporting signal with five ICI monotherapies and two combination therapies represents an important finding given their widespread use in solid tumors. In addition, we also reviewed 13 AA cases induced by ICIs reported in the literature.

According to the 19 clinical trials of ICIs, the frequency of Haem-irAEs induced by ICIs was 3.6% for all grades and 0.7% for grades III-IV, from which aplastic anaemia was considered to be the most serious and life-threatening complication (Michot et al., 2019). Although the specific mechanism of AA-induced by ICIs is not known, the possible mechanism is that ICIs can lead to downregulation of the expression of T cell receptor molecules (primarily CTLA-4 and PD-1), which contribute to an overactive immune response. The inverted ratio of CD4/CD8 indicates inhibitory T cell sensitization (Zhuang et al., 2020). This mechanism was also proved by some cases who did the marrow biopsy from our AA case series (Michot et al., 2017) (Helgadottir et al., 2017) (Meyers et al., 2018) (Younan et al., 2021), which showed activated CD8^+^ lymphocytes that were suggestive of immune-mediated aplastic anaemia.

Both pharmacovigilance results and case series showed that nivolumab and ipilimumab/nivolumab were the leading suspected ICI regimens, and we also identified avelumab, durvalumab and atezolizumab/nivolumab reported ICSRs associated with AA which case series did not. The median age of patients in our pharmacovigilance analysis was 68 years (IQR 26–83) and 67 years (IQR 45–78) for the cases of ICI-induced AA published in the case reports, which is in line with another observational study of 62 years (IQR 52–74) among ICI-induced pancytopenia or AA patients (Delanoy et al., 2019). More than half of AA events occurred in female patients and the indications mainly included lung and skin cancers. Nine of the 13 cases of ICI-induced AA had a fatal outcome (69.2%) and 34.2% of ICSRs from our FAERS analysis were dead, thereby highlighting the severity and life-threatening nature of this potential adverse event. The median time to onset of AA in our study was 34 days, ranging from 10 to 510 days which is similar to time to onset of other classical irAEs (Tang et al., 2021). The time to onset of AA was varied in our study (10–510 days), and most ICSRs were collected within the first 2 months underscoring the significance of closely monitoring AA over the 60 days. Besides, the case series of AA also showed the same trend. Furthermore, seven patients (53.8%) of the AA case series were detected on regular laboratory tests, emphasizing the importance of regular laboratory evaluation especially when patients experiencing symptoms of anemia.

Our study identified a significant signal between different ICI monotherapies and AA, except for ipilimumab and cemiplimab which had no reported AA ICSRs in the FAERS database during the study period. A retrospective study found two hematological events related to ipilimumab: autoimmune haemolytic anaemia (AIHA) and leucopaenia (Kramer et al., 2021), and a systematic review identified 15 ipilimumab-induced hematological events but no AA (Omar et al., 2020). And from another pharmacovigilance study, ipilimumab monotherapy was found significantly associated with autoimmune neutropenia (ROR025 4.22), haemolytic anaemia (2.10), immune thrombocytopenic purpura (ROR025 1.59) and other ten hematological AEs, while neutropenia was the only positive signal for cemiplimab monotherapy among all hematological AEs (ROR025 1.48) (Ye et al., 2020), however, no such clinical cases were reported. We also found ICSRs with AA related to atezolizumab, avelumab, durvalumab and atezolizumab/nivolumab, which were not reported in the literature, emphasizing the significance of signal detecting of FAERS. Noteworthily, this is the first report of such an ICI that could cause AA. Furthermore, of all ICI monotherapies, avelumab had the strongest correlation with AA, and the underlying mechanism for this remains to be discovered. We also found that ICI-associated AA were over-reported for ipilimumab/nivolumab vs nivolumab [ROR 2.57 (1.15, 5.72)]. Besides, a meta-analysis study based on 2,946 patients from four studies also discovered that ipilimumab/nivolumab may result in higher all- and high-grade irAEs compared with nivolumab alone, including pruritus, rash, diarrhea, colitis, alanine aminotransferase elevation, and pneumonitis (Zhou et al., 2019). Therefore, additional pharmacological intervention to prevent or treat these adverse events should be considered when standardizing the combination of anti-PD-1 and anti-CTLA-4 in metastatic tumors.

According to a recently published guideline on the management of immune toxicities, ICIs should be discontinued in the event of grade 1 aplastic anemia (Brahmer et al., 2018), which is also recommended by the general guideline for AA that causative drugs should be discontinued (Killick et al., 2016). And the treatment mainly includes supportive transfusions, growth factors, and immunosuppression such as horse ATG plus cyclosporine and rabbit ATG plus cyclosporine according to the adverse events grading; for patients with grade 2 AA toxicities, HLA typing and evaluation for bone marrow transplantation are recommended. However, since most of the patients from the AA case series were senile, making immunosuppressive therapy was the optional choice. However, only two patients were administered immunosuppressive therapy of cyclosporine or horse ATG plus cyclosporine, but responded poorly and then died (Younan et al., 2021). Besides the above supportive and immunosuppressive treatment for AA, it is well known that corticosteroids can stimulate bone marrow erythropoiesis and reduce excessive menstrual bleeding in women, which are the basic hematopoietic drugs for AA. However, due to unproven benefits and increased risk of infection, corticosteroids are not recommended for treating severe AA (Bacigalupo, 2017). Among 13 ICI-induced AA cases, 76.9% of them were administered steroids, from which 3 got a full recovery and one died of complications of pneumonia (Filetti et al., 2019). Therefore, in most patients with AA-induced by ICIs, corticosteroids plus adequate anti-infective prophylaxis seems a suitable option. The widely used corticosteroids can be explained by their relative availability, low cost, and physicians’ experience compared to other options. However, steroids have an extremely high failure rate for AA-induced by ICIs which implies seeking other treatment options such as immunosuppressive therapy as guidelines recommended and keeping patients on steroids closely monitored. Moreover, eltrombopag has been proposed as a potential option for non-severe AA because of its excellent tolerability and efficacy (Peslak et al., 2017) and is also recommended by the guideline for refractory AA patients (Brahmer et al., 2018). To the best of our knowledge, none of the published cases of ICI-induced AA rechallenged patients with their ICI, which is appropriate given the life-threatening nature of this irAE.

Further attention should be given to AA induced by ICIs randomized controlled trials to provide better quality evidence in this field, and more data is also needed to assess the morbidity and risk factors related to the development of AA. Moreover, it is imperative to elucidate the mechanisms of these adverse events at the molecular and cellular levels, leading to more effective pharmacological treatments. Evidence-based guidelines are needed for the management of AA in patients undergoing ICIs to guide the treatment of such patients.

Our study has several limitations. Firstly, the data mining method cannot offset the innate drawbacks of FAERS including underreporting, false reporting, incomplete reporting, inaccuracy, and so on, all of which might lead to bias. Secondly, qualitative research can only be applied because of the intrinsic nature of FAERS. The signals of AA-induced by ICIs cannot be quantified by the total number of adverse reactions, nor could the incidence of AA be calculated. Thirdly, the ICSRs cannot establish a causal relationship to drug-induced events. Although some genetic limitations of the FAERS database, it marks some key aspects of ICI-associated AA, providing clues for further well-designed studies.

## 5 Conclusion

In conclusion, there is a significant reporting signal of aplastic anaemia with nivolumab, pembrolizumab, durvalumab, atezolizumab, abvelumab, ipilimumab/nivolumab and atezolizumab/nivolumab in the FAERS database, and AA-induced by ICIs including durvalumab, atezolizumab and abvelumab were detected for the first time. One striking finding was that avelumab was more strongly associated with AA than other ICIs, while ipilimumab/nivolumab presented higher reporting than nivolumab. Clinicians should raise awareness and monitor this potentially fatal adverse event. Furthermore, we expect more pharmacovigilance studies, cohort studies and clinical trials in the future to generalize evidence-based treatment strategies for patients with ICIs induced AA.

## Data Availability

The original contributions presented in the study are included in the article/Supplementary Material, further inquiries can be directed to the corresponding author.
