# Supplementary material for: Immune checkpoint inhibitor-induced aplastic anaemia: Case series and large-scale pharmacovigilance analysis
Source: Front Pharmacol. 2023 Jan 26;14:1057134. doi: 10.3389/fphar.2023.1057134 (PMC9908595; doi:10.3389/fphar.2023.1057134)
Supplement: Supplementary file 1 [file Table1.DOCX]

Supplementary Material

Table S1 Clinical characteristics of patients with ICI-associated aplastic anaemia collected from the FAERS database (January 2011 to June 2022).

| Characteristics |  | Fatal cases | Non-fatal cases | P-value |
| --- | --- | --- | --- | --- |
| Gender | Male | 2 (15.4) | 12 (48.0) | 0.159 |
|  | Female | 9 (69.2) | 9 (36.0) |  |
|  | Not reported | 2 (15.4) | 4 (16.0) |  |
| Age | Unknown | 3 (23.1) | 6 (24.0) | 0.353 |
|  | Median | 67 (26-78) | 68 (34-83) |  |
| Reporting year | 2016 | 0 (0) | 3 (12.0) | 0.300 |
|  | 2017 | 1 (7.7) | 1 (4.0) |  |
|  | 2018 | 7 (53.8) | 5 (20.0) |  |
|  | 2019 | 3 (23.1) | 3 (12.0) |  |
|  | 2020 | 0 (0) | 5 (20.0) |  |
|  | 2021 | 0 (0) | 6 (24.0) |  |
|  | 2022 | 2 (15.4) | 2 (8.0) |  |
| Source region | North America | 8 (61.5) | 9 (36.0) | 0.222 |
|  | Europe | 3 (23.1) | 9 (36.0) |  |
|  | Asia | 2 (15.4) | 7 (28.0) |  |
| Indications | Central nervous system tumor | 0 (0) | 3 (12.0) | 0.504 |
|  | Gastrointestinal cancer | 0 (0) | 2 (8.0) |  |
|  | Tumours of the female reproductive organs | 0 (0) | 1 (4.0) |  |
|  | Haematological cancer and lymphoma | 2 (15.4) | 0 (0) |  |
|  | Lung cancer | 4 (30.8) | 8 (32.0) |  |
|  | Skin cancer | 3 (23.1) | 7 (28.0) |  |
|  | Tumours of the urinary system | 1 (7.7) | 1 (4.0) |  |
|  | Unknown or missing | 3 (23.1) | 3 (12.0) |  |
| Outcomes | Death | 13 (100.0) | 0 (0) | <0.0001 |
|  | Hospitalization | 0 (0) | 8 (32.0) |  |
|  | Life-threatening | 0 (0) | 3 (12.0) |  |
|  | Other | 0 (0) | 14 (56.0) |  |
| Drug | Atezolizumab | 1 (7.7) | 1 (4.0) | 0.927 |
|  | Avelumab | 0 (0) | 2 (8.0) |  |
|  | Durvalumab | 0 (0) | 1 (4.0) |  |
|  | Nivolumab | 5 (38.5) | 7 (28.0) |  |
|  | Pembrolizumab | 2 (15.4) | 6 (24.0) |  |
|  | Atezolizumab+nivolumab | 0 (0) | 1 (4.0) |  |
|  | Nivolumab+ipilimumab | 5 (38.5) | 7 (28.0) |  |
| Total |  | 13 | 25 |  |
